# Mecp2 promotes the anti‐inflammatory effect of alpinetin via epigenetic modification crosstalk

**DOI:** 10.1111/jcmm.18510

**Published:** 2024-07-02

**Authors:** Ke Hu, Ruoting Ma, Minjiang Huang, Xiangyu Cao, Yan Ding, Yuxian Li, Yuefu Chen, Lijun Xiao, Sha Ling, Youliang Huang, Huiming Yin, Bifeng Tan

**Affiliations:** ^1^ Medical College Hunan University of Medicine Huaihua China; ^2^ Department of General Medicine, The Third Xiangya Hospital Central South University Changsha China; ^3^ Department of Cardiology, First Affiliated Hospital Hunan University of Medicine Huaihua China; ^4^ Department of Respiratory and Critical Care Medicine, First Affiliated Hospital Hunan University of Medicine Huaihua China

**Keywords:** acetylation, alpinetin, inflammation, methylation, methyl‐CpG‐binding protein 2, P300

## Abstract

In recent years, inflammatory disorders have emerged as a significant concern for human health. Through ongoing research on anti‐inflammatory agents, alpinetin has shown promising anti‐inflammatory properties, including involvement in epigenetic modification pathways. As a crucial regulator of epigenetic modifications, Mecp2 may play a role in modulating the epigenetic effects of alpinetin, potentially impacting its anti‐inflammatory properties. To test this hypothesis, two key components, p65 (a member of NF‐KB family) and p300 (a type of co‐activator), were screened by the expression profiling microarray, which exhibited a strong correlation with the intensity of LPS stimulation in mouse macrophages. Meanwhile, alpinetin demonstrates the anti‐inflammatory properties through its ability to disrupt the synthesis of p65 and its interaction with promoters of inflammatory genes, yet it did not exhibit similar effects on p300. Additionally, Mecp2 can inhibit the binding of p300 by attaching to the methylated inflammatory gene promoter induced by alpinetin, leading to obstacles in promoter acetylation and subsequently impacting the binding of p65, ultimately enhancing the anti‐inflammatory capabilities of alpinetin. Similarly, in a sepsis mouse model, it was observed that homozygotes overexpressing Mecp2 showed a greater reduction in organ damage and improved survival rates compared to heterozygotes when administered by alpinetin. However, blocking the expression of DNA methyltransferase 3A (DNMT3A) resulted in the loss of Mecp2′s anti‐inflammatory assistance. In conclusion, Mecp2 may augment the anti‐inflammatory effects of alpinetin through epigenetic ‘crosstalk’, highlighting the potential efficacy of a combined therapeutic strategy involving Mecp2 and alpinetin for anti‐inflammatory intervention.

## 1 INTRODUCTION

Inflammation is critical for survival during physical injury and infection. Yet, excessive synthesis and release of inflammatory mediators may damage organ function. Prolonged chronic inflammation can increase the risk of heart disease, diabetes,^1^ cancer^2^ and rheumatoid arthritis.^3^ Nonsteroidal anti‐inflammatory drugs and steroid injections are commonly used to treat severe inflammation but also have certain side effects.^4^, ^5^ Recent studies have been focusing on investigating small molecular inhibitors targeting nuclear factor kappa‐B (NF‐κB),^6^ mitogen‐activated protein kinases,^7^ Toll‐like receptors,^8^ or other members of the inflammatory pathways^9^; however, no breakthrough has been achieved. Thus, exploring intervention strategies with significant anti‐inflammatory effects and low toxic side effects is needed.

Alpinetin is a traditional Chinese drug isolated from the plant *Alpinia katsumadai Hayata*, which has anti‐bacterial, anti‐oxidant, and anti‐inflammatory effects.^10^, ^11^ In a previous study, we assessed the anti‐inflammatory effect of alpinetin and confirmed that this drug could inhibit the expression of NF‐κB and extracellular signal‐regulated kinase family members through the peroxisome proliferator‐activated receptors (PPAR), affecting the expression of *IL‐6* and *TNF‐α*.^12^, ^13^ Moreover, we found that PPAR activation induced by alpinetin can further promote the synthesis and modification activity of DNA methyltransferase DNMT3A in the nucleus of RAW 246.7 (the macrophage cell line), thus promoting the methylated modification of the IL‐6 promoter and blocked the binding of transcription factors. Therefore, the PPAR/DNMT3A pathway is considered the main molecular mechanism involved in reversing inflammatory organ damage by alpinetin.^14^, ^15^

Methyl‐CpG‐binding protein 2 (Mecp2) is a crucial transcriptional regulator for synaptic function that binds to methylated DNA.^16^ Our previous study confirmed that Mecp2 could inhibit the binding of the coactivator p300 to the methylated IL‐6 promoter in human embryonic kidney cells. We also confirmed the important role of p300 in initiating the inflammatory expression process, that is, by combining with the promoter site of the inflammatory gene, acetylation of H3K27 in this region occurs, which loosens the structure of chromatin, thus facilitating the binding of transcription factors.^17^ Additionally, Piazza suggested that Mecp2, as well as Sin3 transcription regulator family member A (Sin3A) and histone deacetylase 1 (HDAC1), can form fusion proteins, influencing changes in chromatin structure and interfering with gene expression, which further support that Mecp2 can regulate acetylation modification of histones.^18^ All this data suggests that Mecp2 is likely to engage in the form of communication known as ‘crosstalk’ with the acetylated modification facilitated by p300 and the methylated modification induced by alpinetin during the regulation of inflammatory gene expression, which may ultimately enhance the anti‐inflammatory efficacy of alpinetin.

In this study, mouse macrophages and gene‐edited mice overexpressing Mecp2 were used to validate the above hypothesis and provide initial insights into the molecular mechanism underlying the facilitation of alpinetin's anti‐inflammatory effect caused by Mecp2.

## 2 METHODS

### 2.1 Cell culture

RAW 246.7 cells (Nanjing Huaao Biotechnology Co., Ltd., China) were cultured in Roswell Park Memorial Institute (RPMI) 1640 medium (Promega Corporation, USA) containing 10% fetal bovine serum (FBS, Promega Corporation, USA) and 1% penicillin/streptomycin (Promega Corporation, USA) in a humidified atmosphere containing 5%CO_2_/95% air at 37°C. Cells were cultured for a few passages before being used in the experiments.

### 2.2 Screening of moleculars related to LPS stimulation intensity

The Agilent Sureprint G3 Mouse Gene Expression v2 Microarray (8 × 60K) was collaboratively developed by Agilent and the John Rinn laboratory, encompassing 39,430 Entrez Gene RNAs and 16,251 lincRNAs. RAW264.7 cells were revived and cultured in RPMI‐1640, then categorized into four groups based on varying concentrations and durations of LPS (*Escherichia coli* 055:B5) stimulation: (1) 1 μg/mL LPS for 6 h; (2) 10 μg/mL LPS for 12 h; (3) 100 μg/mL LPS for 12 h; (4) Control group: receiving an equivalent volume of medium. After stimulation, cells with viability exceeding 80% (assessed by MTT assay) were selected for chip hybridization experiments. The primary procedures included total RNA purification post‐extraction, cDNA reverse transcription, cRNA transcription and synthesis, sample fragmentation for fluorescence labeling, and chip hybridization (rolling at 65°C for 10 r/min for 18 h). Subsequently, washing and scanning were conducted to capture the hybridization image. Next, datas were extracted utilizing Feature Extraction software post‐scanning and normalized using Genespring.

### 2.3 Construction of RAW 246.7 cells overexpressing Mecp2

The coding sequence (CDS) of mouse Mecp2 protein (1077 bp) was obtained from the NCBI official website (https://www.ncbi.nlm.nih.gov), and the primer sequences were designed based on this template. The structural composition of the overexpression vector was protective sequence+restriction endonuclease site+20 bases, respectively located at the head and tail ends of the CDS sequence (upstream primer sequence: 5′‐ATGGTAGCTGGGATGT‐TAGG‐3′; downstream primer sequence: 5′‐GTGACCGAGAGAGTTAGCTG‐3′). The control vector P‐EGFP‐basic was constructed using the head and tail ends of the intron fragments as the template, which has the same length as the amplified region of the overexpression vector. Both vectors were synthesized and assembled by Wuhan Biotechnology Co., Ltd.

RAW 246.7 cells were cultured on a WellReady™ plate (Atelerix, WR‐Z384). After reaching 80% confluence, 2 μg of recombinant plasmid (1 mmol/L) and 4 μL of Lipofectamine 2000 (Thermo Fisher Scientific, 11668027) were mixed in 50 μL of Opti‐MEM medium (Promega Corporation, USA). After 24 h, more than 90% of the transfected cells expressed green fluorescence, which was visible under the microscope (Zeiss, Germany).

### 2.4 Cell grouping and intervention

The high‐purity alpinetin extract (≥99.5%) was purchased from Nanjing Zelan Biotechnology Co., Ltd. RAW 246.7 cells transfected with or without Mecp2 overexpression plasmid were further divided into the following eight groups: (1) control group; (2) 250 μg/mL alpinetin group; (3) 500 μg/mL alpinetin group; (4) 1 mg/mL alpinetin group; (5) 250 μg/mL alpinetin+DNMT3A interference group; (6) 500 μg/mL alpinetin+DNMT3A interference group; (7) 1 mg/mL alpinetin+DNMT3A interference group; (8) 500 μg/mL alpinetin+negative interference group. DNMT3A interference was conducted by interfering plasmid (fragment: 5′‐CGGACCACCTTACGTGACC‐3′, constructed by Wuhan Biotechnology Co., Ltd.).

### 2.5 Detection of CpG dinucleotide methylation level in IL‐6 promoter region by bisulfite sequencing PCR (BSP)

UCSC Genome Browser (http://genome.ucsc.edu/) and PROMO website, a transcription factor binding site identification tool (http://alggen.lsi.upc.es/cgi‐bin/‐promo v3/promo/promoi‐nit.cgi?dirDB = TF8.3), were used to predict the transcription factor binding sites on mouse inflammatory genes IL‐6 and TNF‐α. Leverage Cpgplot (http://emboss.Bioinfor‐ matics.nl/cgi‐bin/‐Emboss/cpgplot) software was used to evaluate whether the promoter regions of IL‐6 and TNF‐α contain fragments that meet the definition of the CpG island.

Then, the BSP method was performed. First, 5 × 10^5^ cells from each group were selected. The DNA was then extracted using the Wizard genome purification kit (Promega Company, USA), and a sodium bisulfite modification was performed using the Metrology Gold™ kit (Promega Company, USA) following the manufacturer's instructions. The amplification upstream and downstream primers (5′‐ATCTGGGAAGATTCCAATT‐3′ and 5′‐CAAAGG‐AGTCATTCGTCTCAT‐3′; the amplification region was located on the CpG island) were designed by the Methyl Primer Express V2.0 software. The total PCR reaction was performed as follows: 50 μL master mix including 1 μL sodium bisulfite modified DNA template, 1 μL upstream and downstream primer each, 5 μL of 10× contains Mg^2+^ PCR buffer, 1.2 μL dNTP and 0.8 μL Taq DNA polymerase. The following conditions were applied: pre‐denaturation at 95°C for 5 min, post‐denaturation at 94°C for 35 s, annealing at 55°C for 35 s, and extension at 72°C for 30 s, for a total of 40 cycles. Finally, the products were sent to Shenzhen Huada Genomics (China) for time‐of‐flight mass spectrometry (TOF‐MS) sequencing. According to the modification principle, if cytosine was methylated, the sequencing result was still cytosine (C); if not, the result was thymine (T). Each site was detected three times, and the CpG sites within a certain island range were arranged in order. Colour labeling was carried out according to the following methods: if all the results were thymine, the colour was white; if the test result was cytosine, it was marked as light brown, dark brown, and black according to the frequency of one, two, and three times, and the methylation modification ratio was calculated accordingly.

### 2.6 Western blot (WB)

The proteins were lysed using the RLN lysis buffer containing 0.1 mol/L Tris–HCl, 150 mmol/L NaCl, 1.5 mmol/L MgCl_2_, and 0.5% Nonidet. Samples were separated by 10% SDS‐PAGE electrophoresis and then electrotransferred on a PVDF membrane in an ice bath. Then, the membrane was blocked in 5% skim milk at room temperature for 2 h. Next, the membrane was incubated with primary antibodies (Cell Signalling Technology, USA; diluted at 1:2000), targeting p65, p300, Mecp2, DNMT1, DNMT3A, GAPDH and β‐actin, at 4°C overnight. After that, samples were washed with TBST three times and incubated with HRP‐labelled secondary antibodies (Beijing Boda Tech, diluted at 1:1000) at room temperature for 2 h. Enhanced chemiluminescence (ECL) imaging was performed after washing. AlphaEase FC Version 4 software was used to detect the absorbance (A) value of protein bands.

### 2.7 Enzyme‐linked immunosorbent assay (ELISA)

IL‐6 and TNF‐α Mass concentration medium were detected by ELISA (Beijing Boda Tech, China) following the manufacturer's instruction.

### 2.8 CHIP‐Seq

CHIP‐Seq was used to assess acetylated H3K27 modification of the TSS upstream region of the IL‐6 gene and the p65, p300 and Mecp2 binding levels. Briefly, RAW 264.7 cells (5 × 10^5^) were fixed using formaldehyde, treated with glycine, shaken at room temperature for 5 min, centrifuged at 4°C, and washed with precooled PBS. The samples were then mixed with 1 mL of buffer‐containing inhibitor and incubated on ice for 10 min. Ice ultrasound (power set at 50 W, eight times in total, 1 min each time, 1 min interval) was used to break DNA fragments to 100–500 bp. The samples were centrifuged at 4°C and 14,000 r/min for 10 min, and the supernatant was collected. The samples were divided into two tubes; one was mixed with 2 μg IgG antibody used as the chip sample, while 2 μg rabbit anti‐mouse p65, p300, Mecp2 or acetylated H3K27 monoclonal antibody (all diluted at 1:1000, ab263899, Abcam) were added to the other tube and incubated at 4°C overnight. Then, the samples were centrifuged at 14,000 r/min for 15 min, and the supernatant was discarded. The samples were washed with elution buffer, oscillated for 15 min, and centrifuged for 10 min at 14,000 r/min, after which the supernatant was collected. The DNA rapid purification kit (Promega Company of the United States) was used for purification, and the DNA concentration was detected using a Thermofly 200 ultramicro spectrophotometer. The 100–200 bp product was screened, and the 3′ end was connected with base A and the sequencing connector. High‐throughput sequencing was performed by Shanghai Ouyi Biomedical Technology Co., Ltd. CisGenome v2.0 software formatted the sequencing data into a wig file and uploaded it to the IGV_2.9.4 system for data visualization. Mouse_mm10 was set as the comparative genome, and the core analysis region was the binding site predicted by PROMO and its nearby range. Data (reads) with a mismatch of ≤2 bases were used; finally, the image results of p65, p300, Mecp2 and acetylated H3K27 binding peak were obtained.

### 2.9 Mecp2 KI gene editing mice

The animal experiments were approved by the Animal Experiment Ethics Committee of Hunan University of Medicine (approval number: 2020‐HUOM0004). All animal experiments were carried out in the Experimental Animal Center of Central South University. All animal studies (including the mice euthanasia procedure) were done in compliance with the regulations and guidelines of Central South University institutional animal care and conducted according to the AAALAC and the IACUC guidelines.

The Mecp2 KI Gene Editing Mice (background: C57BL/6, male, about 9–10 weeks old) were purchased from Cyagen Biosciences Inc. (Suzhou, China). After Southern blot, genotypes of 120 homozygous were identified as ROSA26_Mecp2 (CKI/CKI), CAGGCre‐ERTM; 55 heterozygous were identified as ROSA26_Mecp2 (CKI/+), CAGGCre‐ERTM; another 65 were ROSA26_Mecp2 (CKI/+), Cre negative. Blood (0.1 mL) was collected from the tail vein. After centrifugation for half an hour at 500 rpm, mononuclear and neutrophil layers in the blood were separated. Then, the total protein was extracted, and the expression level of Mecp2 was detected by WB.

Consequently, three animals were randomly selected from the heterozygotes and homozygotes for the open field test (OFT), which was used to assess locomotor activity and anxiety‐like behaviour. All subjects were housed in an environment with a temperature of 22 ± 1°C, relative humidity of 50 ± 1%, and a light/dark cycle of 12/12 h for 1 week before the test. The OFT testing was performed as previously described,^19^ and the OFT apparatus consisted of a large arena measuring 120 × 120 × 60 cm (*L* × *W* × *H*). The arena was made of black high‐density polyethylene panels placed on a plastic bottom plate. A Nolvasan solution was applied as a regular disinfectant (Faulkner Plastics, Miami, FL). The activity area consisted of two parts, that is, an outside zone (120 cm wide) and a center zone (60 cm wide). The behaviour of mice was observed using an overhead camera positioned above the arena for 15 min. The EthoVision XT 11.5 software (Noldus Information Technology b.v) was employed to analyse the total distance covered by mice and the frequency of entries into the central zone.

### 2.10 Animal model

The homozygous and heterozygous objects were assigned to six groups randomly (with 20 mice each): (1) control group; (2) cecal ligation and perforation (CLP) group; (3) CLP+alpinetin (12.5 mg/kg) group; (4) CLP+alpinetin (25 mg/kg) group; (5) CLP+alpinetin (50 mg/kg) group; (6) CLP+alpinetin (50 mg/kg)+DNMT3A interference group. The CLP modelling process was conducted as follows: first, the mice were fasted for 12 h before operation, after which 5% chloral hydrate (0.3 mL/100 g) was intraperitoneally injected. Next, an incision of about 2 cm on the abdominal wall was performed under sterile conditions, separating the distal end of the ileocecal valve and ligating its proximal 1/3 with silk thread. After an injection needle (size 18) punctured the distal end of the ligation, the suture was performed. In the control group, we only placed silk thread in the lower segment of the cecum and closed the abdominal cavity within half an hour.

**FIGURE 1:**
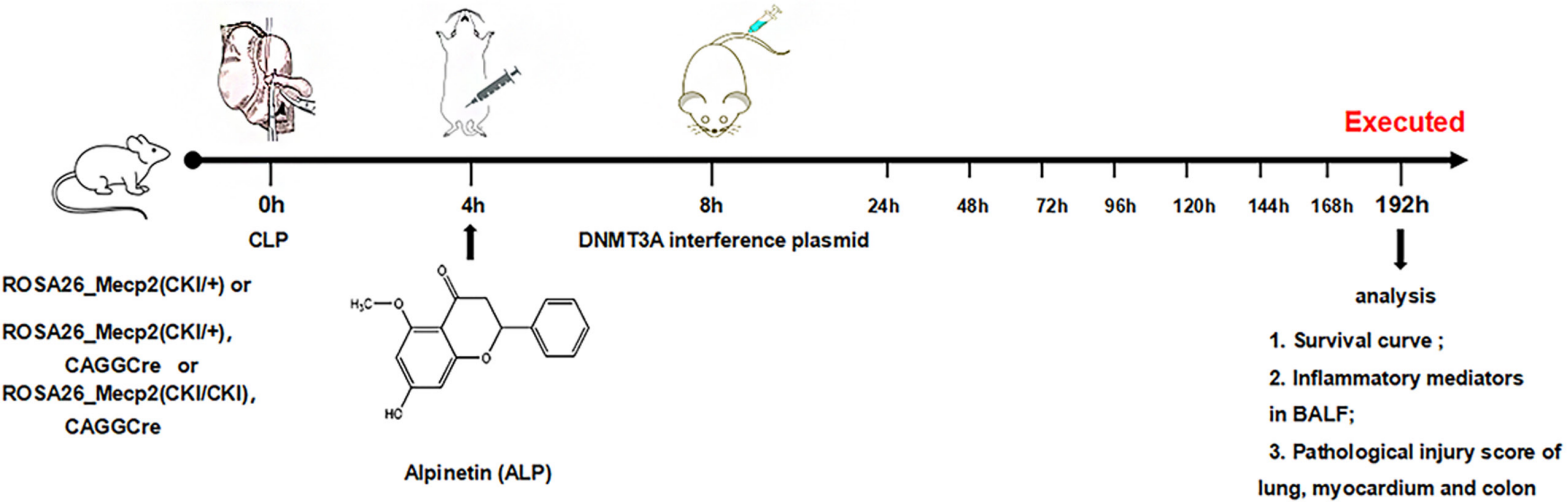
The animal experiment procedure. The animal experiment protocol entailed exposing both individuals with overexpression homozygotes and heterozygotes to either cecal ligation and puncture (CLP) modelling or sham surgery. Subsequently, 4 h post‐procedure, the subjects received intraperitoneal injections of alpinetin at varying doses (12.5–50 mg/kg), followed by intravenous administration of DNMT3A interference plasmid (loading: 10^8^) 8 h post‐procedure. Subjects who did not receive alpinetin or DNMT3A interference were instead injected with an equivalent volume of normal saline (NS) at the corresponding time points. Subsequently, the survival status of the mice was monitored at 24‐h intervals, and the lung, heart and colon tissues of the surviving subjects were harvested at 192 h post‐modelling. Following haematoxylin and eosin staining, pathological alterations were assessed; and the levels of inflammatory mediators in alveolar lavage fluid were quantified.

Four hours after modelling, the mice were given an intraperitoneal injection of alpinetin, while interference plasmid (200 μL, number of virus copies: 10^8^) was injected into the caudal vein 8 h after modelling. For control groups, the same volume of normal saline (NS) was injected at the corresponding time point. The condition of mice was recorded every 24 h until 192 h post‐modelling (Figure 1).

### 2.11 Assessment of inflammatory markers

At the end of the in vivo experiment, mice were intraperitoneally injected with sodium pentobarbital (Zizheng, Shanghai, China) (60 mg/kg). Next, the chest was quickly opened, and a small incision on the left atrial appendage was performed. Then, 250 mL NS was infused from the right ventricle. The lung, heart and colon were dissected and soaked in 4% paraformaldehyde for 24 h, then dehydrated with 300 mL/L sucrose solution and embedded in paraffin. Sections of 5 μm thickness were affixed to slides and stained with H&E to observe histological change. Three random visual fields were selected for each specimen, and pathological scoring was performed based on previous descriptions.^20^ The severity of lung injury was determined by reference to the following indicators: pulmonary congestion, pulmonary haemorrhage, neutrophil infiltration, and alveolar wall thickness (1 point for each item, totaling 4 points). The score of myocardial injury was assessed based on the following criteria: number of infiltrating lymphocytes in the stroma and degree of disordered arrangement of myocardial cells (2 points for each item, totaling 4 points). In addition, Dieleman standard was used to evaluate the colonic mucosal injury index (CMDI), which was scored according to the following four indicators: submucosal edema, loss of goblet cells, PMN infiltration into lamina propria and discontinuity of epithelial layer (1 point for each item, totaling 4 points). Scores of 0%, 20%, 50% and 100% represent completely normal, mild, moderate and significant changes, respectively.

The other side of the lung tissue was ligated at the proximal end, where a scalp needle was inserted and fixed. PBS was slowly pushed until the lung was dilated, then pumped back 30s later. After repeating the above steps several times, alveolar lavage fluid (BALF) was obtained. After centrifugation at 3000 rpm for 5 min, the supernatant of BALF was collected, and the level of IL‐6 and TNF‐α was detected by ELISA.

### 2.12 Statistical analysis

The measurement data of the normal distribution were measured three times, and the results were expressed as mean ± standard deviation. One‐way ANOVA was used for comparison among multiple groups; the least significant difference (LSD) method was used to compare two groups, and the Kaplan–Meier method was used to compare the mouse survival curve. IBM SPSS Statistics 25 software was used for data analysis; *p* < 0.05 was considered statistically significant.

## 3 RESULTS

### 3.1 The expression of p65 and p300 is significantly correlated with the intensity of LPS stimulation in RAW 264.7 cells

**FIGURE 2:**
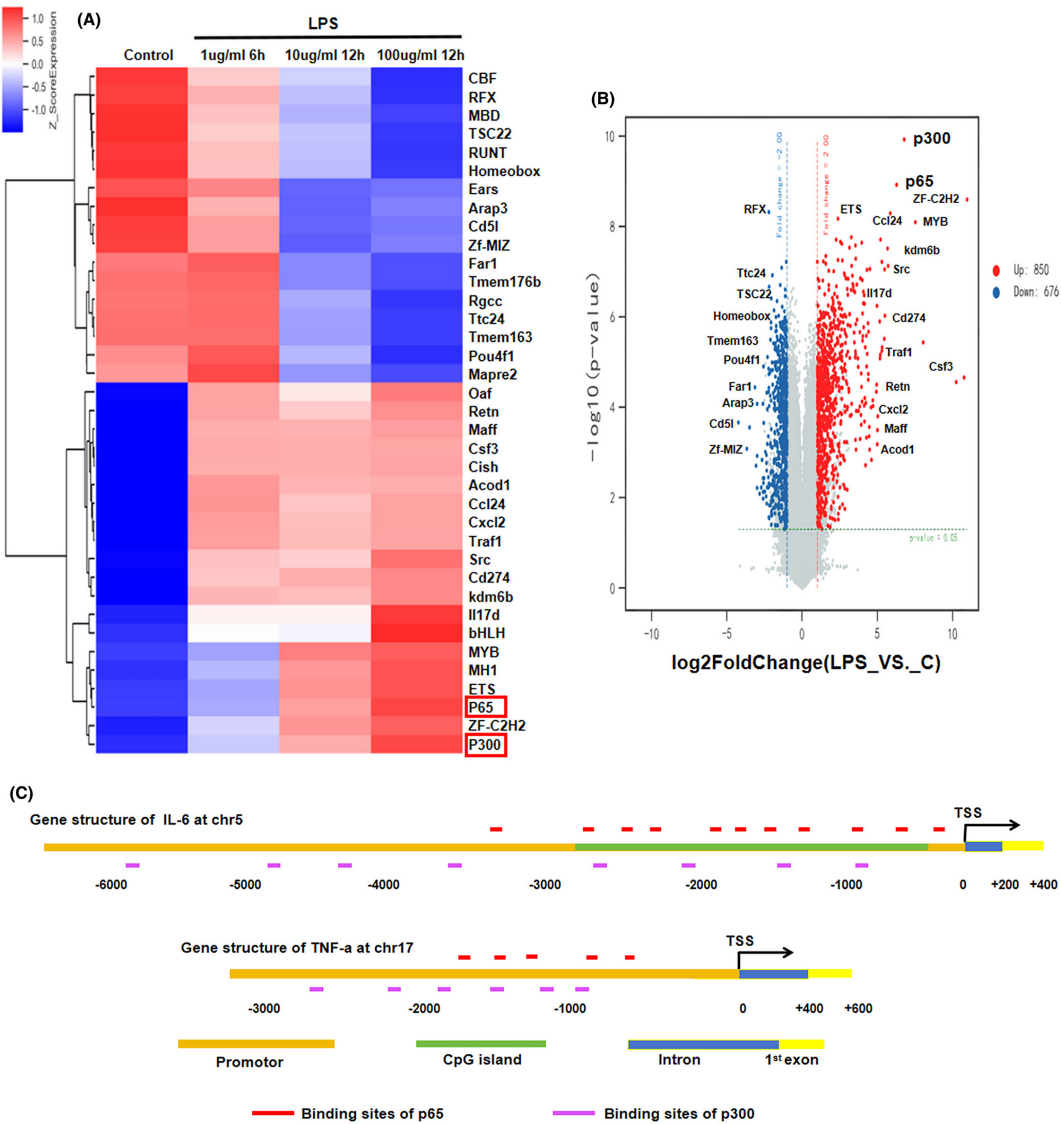
The involvement of p65 and p300 as crucial molecules in response to LPS stimulation was validated through mouse expression profiling chip. (A) The cluster heat map depicting differential gene expression; (B) The volcano plot illustrating expression levels of DEGs; (C) the prediction outcomes from UCSC, PROMO and Cpgplot databases regarding the transcription factor binding sites and CpG islands near the transcription start sites of mouse IL‐6 and TNF‐a.

Genes exhibiting mRNA expression variances of ≥2‐fold with a statistical difference of *p* < 0.05 were categorized as differentially expressed genes (DEGs). As shown in Figure 2A, B, a total of 850 genes were found to be up‐regulated and 676 genes down‐regulated in RAW246.7 cells following LPS stimulation. Notably, the transcription factors p65 and p300, belonging to the nuclear factor‐kappa B (NF‐KB) and co‐activator family respectively, were identified as key members among the DEGs. Furthermore, their expression levels exhibited a consistent correlation with the intensity of LPS stimulation, in addition to the observed increase in synthesis. Moreover, the prediction results of the PROMO website suggest that the promoter regions of the mouse inflammatory genes IL‐6 and TNF‐a both have a high density of p65 and p300 binding sites, which indicates that the transcription of inflammatory genes is mainly driven by these two factors (Figure 2C).

### 3.2 Mecp2 enhances the efficacy of alpinetin in modulating the production of inflammatory mediators in RAW 264.7 cells

**FIGURE 3:**
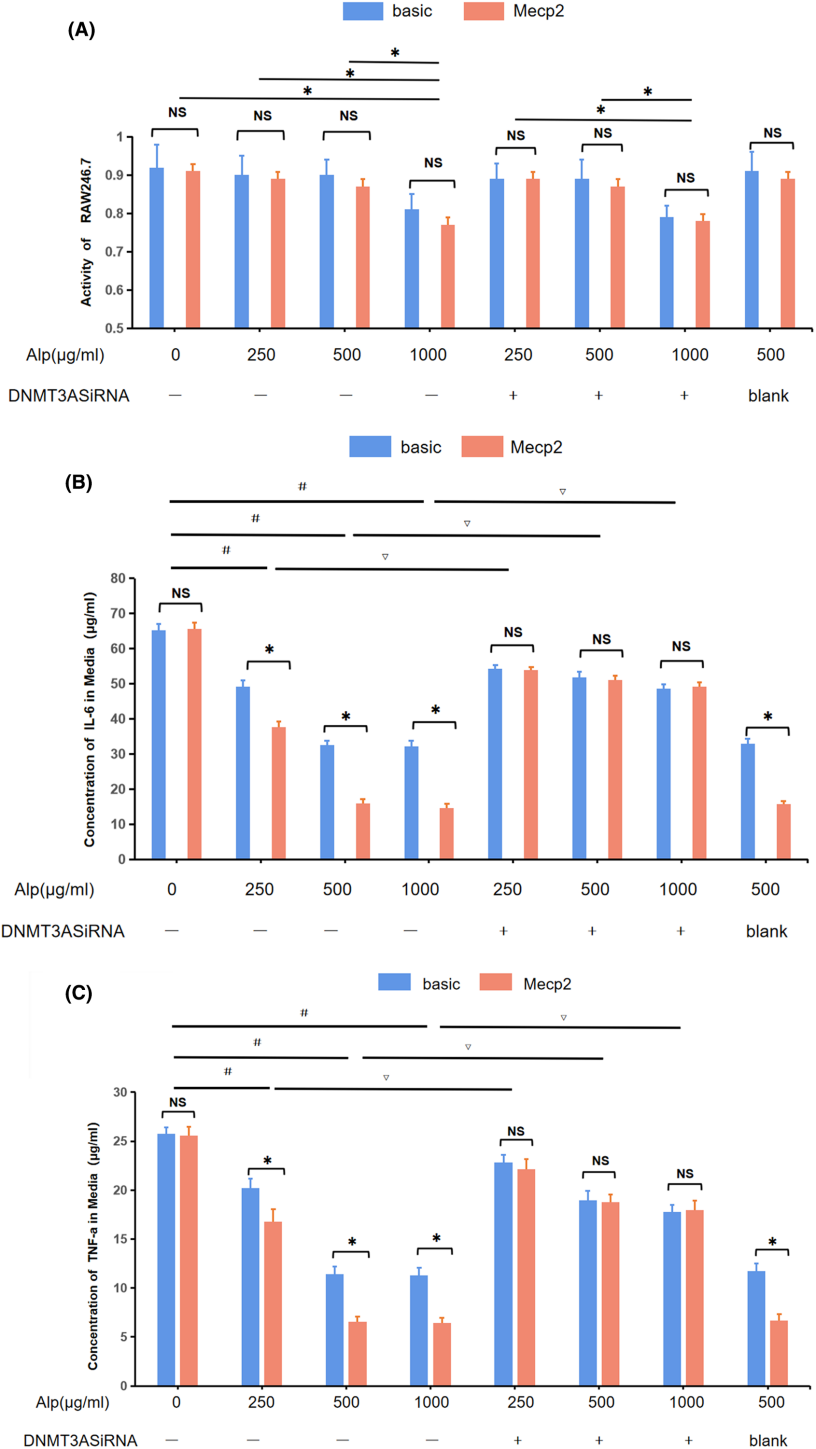
The effect of alpinetin, Mecp2‐overexpressing plasmid and DNMT3A inhibitor on RAW 264.7 cells and its synthetic inflammatory mediators in vitro. (A) Cell viability measured using MTT assay. *p < 0.05 versus control or intervention group of alpinetin under other concentration conditions. (B, C) The expression of IL‐6 and TNF‐α in RAW 264.7 with or without overexpression of Mecp2. ^#^ *p* < 0.05 versus control; ^▽^ *p* < 0.05 versus the groups lacking DNMT3A interference subjected to the same concentration of alpinetin; *p < 0.05 versus the synthesis level in RAW 246.7 not overexpressing Mecp2 under various alpinetin concetrations. Basic: control vector transfection group; Mecp2: Mecp2‐overexpression vector transfection group.

The cytotoxic effect of alpinetin and Mecp2‐overexpressing plasmid were evaluated by the MTT assay. The result showed when the concentration of alpinetin was below 500 μg/mL, the cell viability was not affected. Also, the overexpression of Mecp2 in RAW 264.7 cells did not affect cell viability (Figure 3A). In addition, regardless of Mecp2 overexpression, alpinetin inhibited the synthesis of IL‐6 and TNF‐a in RAW246.7. However, when comparing the concentration of inflammatory mediators in RAW246.7 medium overexpressing Mecp2 to the non‐overexpressing group, the former exhibited significantly lower levels under the same concentration of alpinetin. In addition, we found that the inhibitory effect of alpinetin on the synthesis of inflammatory mediators was partially reversed when DNMT3A was inhibited. Moreover, there was no discernible difference in the synthesis of inflammatory mediators between RAW246.7 overexpression Mecp2 and non‐overexpressing cells in this scenario (Figure 3B,C).

In the subsequent investigation, our focus was solely on elucidating the mechanism at the combination of 500 μg/mL of alpinetin and DNMT3A interference, without delving into the effects at elevated concentrations. This decision was informed by the observed impact of 1 mg/mL of alpinetin on cell viability, as well as the lack of additional inhibition of inflammatory mediator synthesis compared to the 500 μg/mL concentration.

### 3.3 Alpinetin promotes methylation modification of il‐6 promoter through DNMT3A activation in RAW 264.7 cells

**FIGURE 4:**
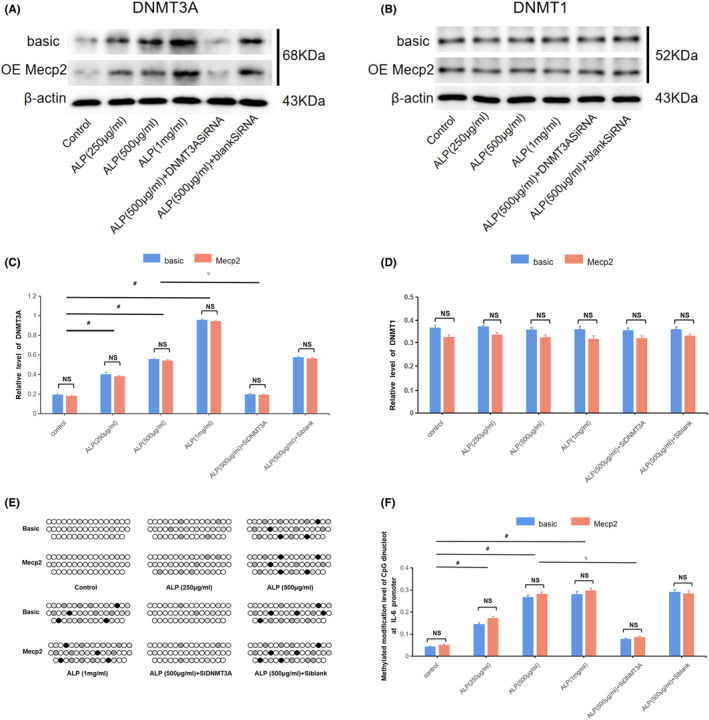
Alpinetin promotes methylation modification of IL‐6 promoter in a dose‐dependent manner through DNMT3A activation in RAW 264.7 cells. (A, B) The detection results of methyltransferase expression in the nucleus of RAW 264.7 by western blot (WB). (C, D) Relative quantitative level of DNMT3A and DNMT1. ^#^ *p* < 0.05 versus control, as the intervention concentration gradually increased. ^▽^ *p* < 0.01 versus 500 μg/mL alpinetin group. (E, F) The methylated modification level of RAW 264.7 inflammatory gene IL‐6 promoter detected by the BSP method. ^#^ *p* < 0.05 versus control, as the intervention concentration gradually increased; ^▽^ *p* < 0.05 versus 500 μg/mL alpinetin group. Basic or Mecp2: RAW 264.7 cells transfected with control or Mecp2 overexpression vectors, respectively.

Firstly, it was confirmed by Cpgplot that the range from 300 to 2500 bp upstream of the IL‐6 transcription initiation site met the definition of a CpG island (Figure 2C). Next, As depicted in Figure 4A–D, alpinetin promoted DNMT3A synthesis in RAW 246.7 nucleus in a dose‐dependent manner but had no significant effect on the synthesis of DNMT1. Furthermore, alpinetin promoted the cytosine methylation level of the IL‐6 promoter, while this effect was reversed after DNMT3A interference. Moreover, Mecp2 overexpression did not affect the methylating effect of alpinetin (Figure 4E,F). Overall, alpinetin promotes methylation modification of IL‐6 promoter through DNMT3A in RAW 264.7 cells.

### 3.4 The binding of Mecp2 to the methylation‐modified promoter site of the IL‐6 gene interferes with p300 binding, assisting the anti‐inflammatory effect of alpinetin

**FIGURE 5:**
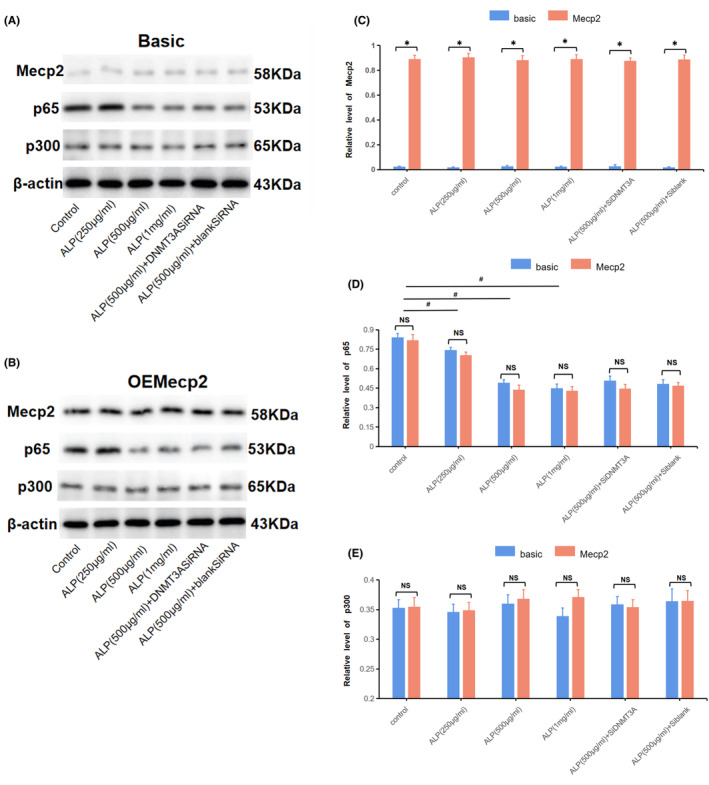
Intranuclear synthesis levels of p65, p300 and Mecp2 in each intervention group. (A, B) Results of p65, p300 and Mecp2 in RAW 264.7 cells with or without overexpression of Mecp2 tested by WB. (C–E)The relative contents of Mecp2, p65 and p300 in the nucleus of RAW 264.7 cells under various intervention conditions: *p < 0.05 versus groups not overexpressing Mecp2 under various intervention conditions (C); ^#^ *p* < 0.05 versus control (D). Basic or Mecp2: RAW 264.7 transfected with control or Mecp2‐overexpression vectors, respectively.

**FIGURE 6:**
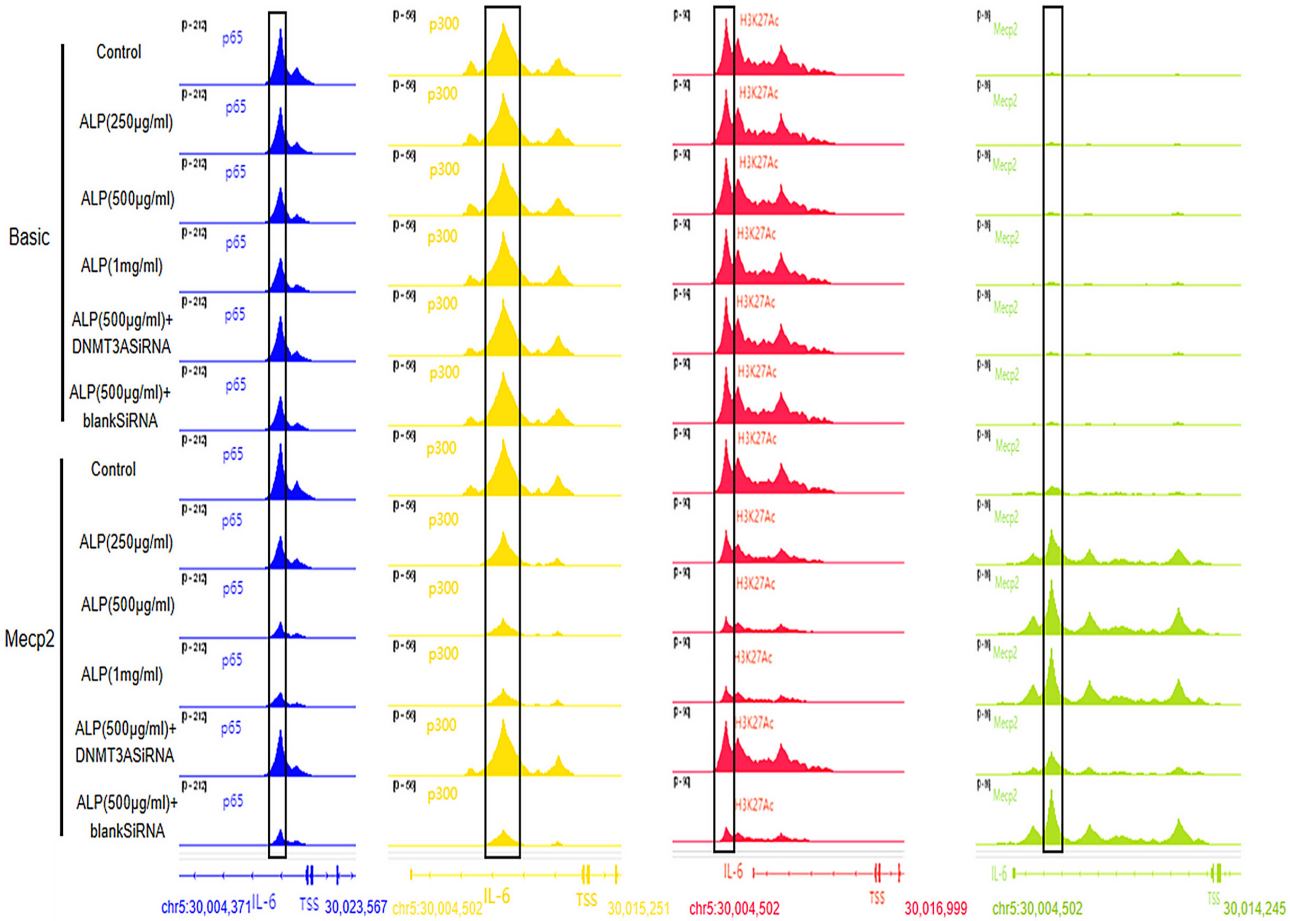
Chip‐seq reveals the molecular mechanism of Mecp2 promoting the anti‐inflammatory effect of alpinetin. Blue, yellow and green represent the combination of p65, p300 and Mecp2 in the corresponding region, respectively; red represents the acetylated level of H3K27 in the corresponding region; the black box marks the comparison region. The above peaks represent the binding level of p65, p300, Mecp2 and the acetylated level of H3K27 in the range from 6000 bp upstream to 1000 bp downstream near the TSS of IL‐6 gene on mouse chromosome 5. Results show that alpinetin can promote the binding of Mecp2 to the promotor of the IL‐6 gene, while DNMT3A interference can prevent this process. The binding level of p300 located in the corresponding region and its related acetylated effect of H3K27 showed an opposite trend with Mecp2. Basic or Mecp2: RAW 264.7 transfected with control or Mecp2‐overexpression vectors, respectively.

As depicted in Figure 5A–C, the Mecp2‐overexpressing plasmid could stably express related products after transfection, which was not interfered by alpinetin. In addition, alpinetin significantly inhibited the synthesis of p65 (Figure 5A,D); however, the synthesis of p300 was not affected by alpinetin or overexpression of Mecp2 (Figure 5A, B, E). In addition, the Chip‐seq analysis (Figure 6)revealed that alpinetin‐induced methylation modification impeded the interaction between p65 and the IL‐6 promoter region; however, this modification did not hinder the binding of p300 to the same region. Conversely, the binding of overexpressed Mecp2 to the methylated IL‐6 promoter consequently inhibited the association of p300 and impeded the acetylation modification of H3K27, which hindered the subsequent binding of p65 and further affected the synthesis of IL‐6.

### 3.5 Mecp2 Further improves the multiple organ damage of sepsis via ‘crosstalk’ of epigenetic modification

**FIGURE 7:**
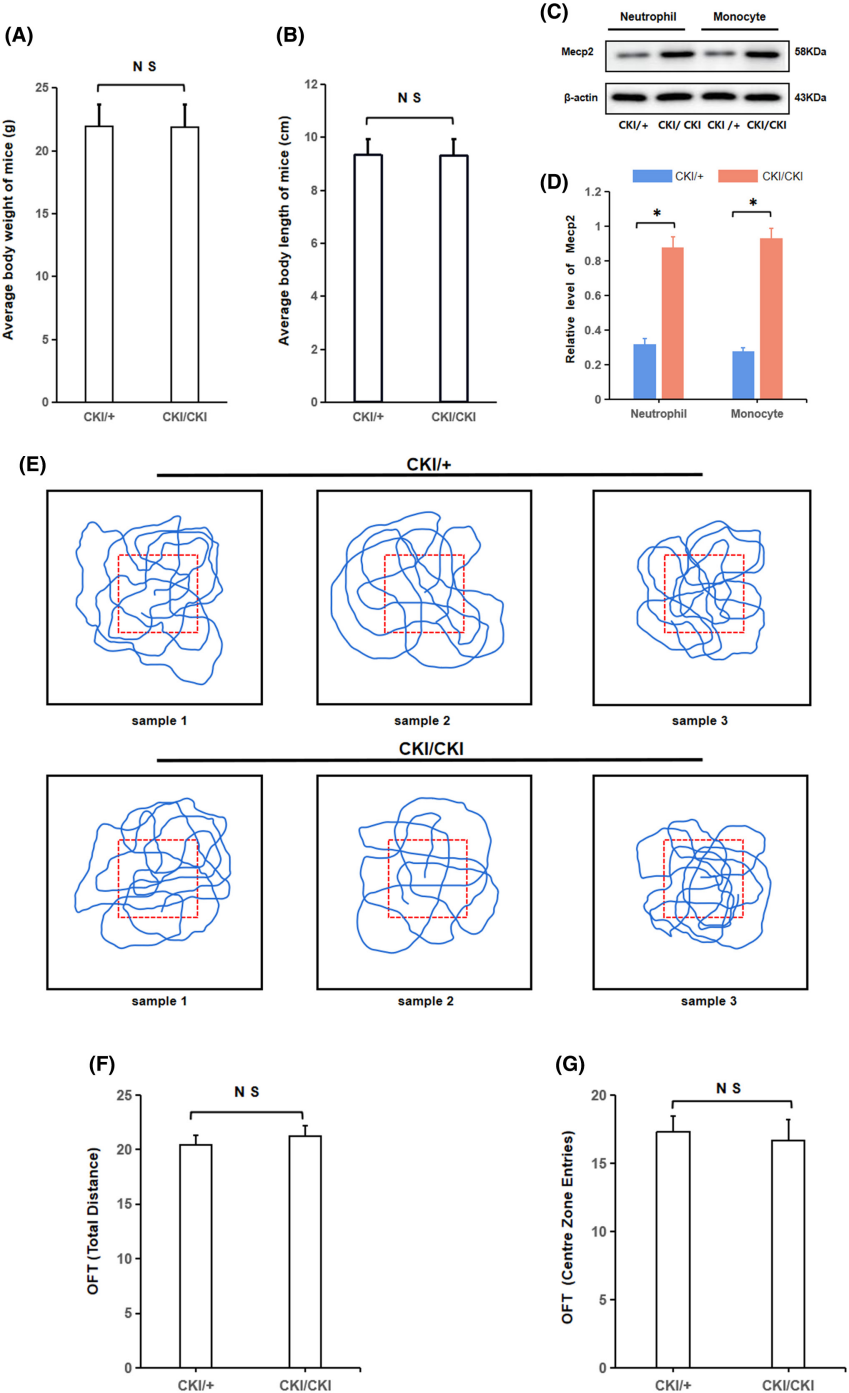
Mecp2 can be consistently expressed in peripheral blood cells of genetically modified mice without significantly affecting mice growth and behaviour. (A, B) Average body weight and length of heterozygous and homozygous mice (9–10 weeks old, *n* = 120). (C) WB detection of Mecp2 in the peripheral blood cells. (D) The relative content of Mecp2 in the peripheral blood cells of heterozygous and homozygous mice: *n* = 3, *p < 0.05 versus Mecp2 (CKI/+) objects. (E) The comprehensive trajectory of the mice captured in photographs during the 15‐min OFT. (F) The mean distance covered by heterozygous and homozygous mice throughout the OFT. (G) the mean frequency at which the heterozygous and homozygous mice crossed the central field throughout the course of OFT.

As depicted in Figure 7A,B, Mecp2 overexpression did not significantly affect the growth and development of animals. Compared with heterozygous, the synthesis of Mecp2 was significantly different in homozygous, which indicates that the knock‐in Mecp2 can stably express in murine immune cells (Figure 7C,D). Furthermore, OFT testing showed no statistically significant distinction in cognitive and function behaviour (walking distance and the number of times they traversed the middle region within a specified time frame) between heterozygous and homozygous mice (Figure 7E–G).

**FIGURE 8:**
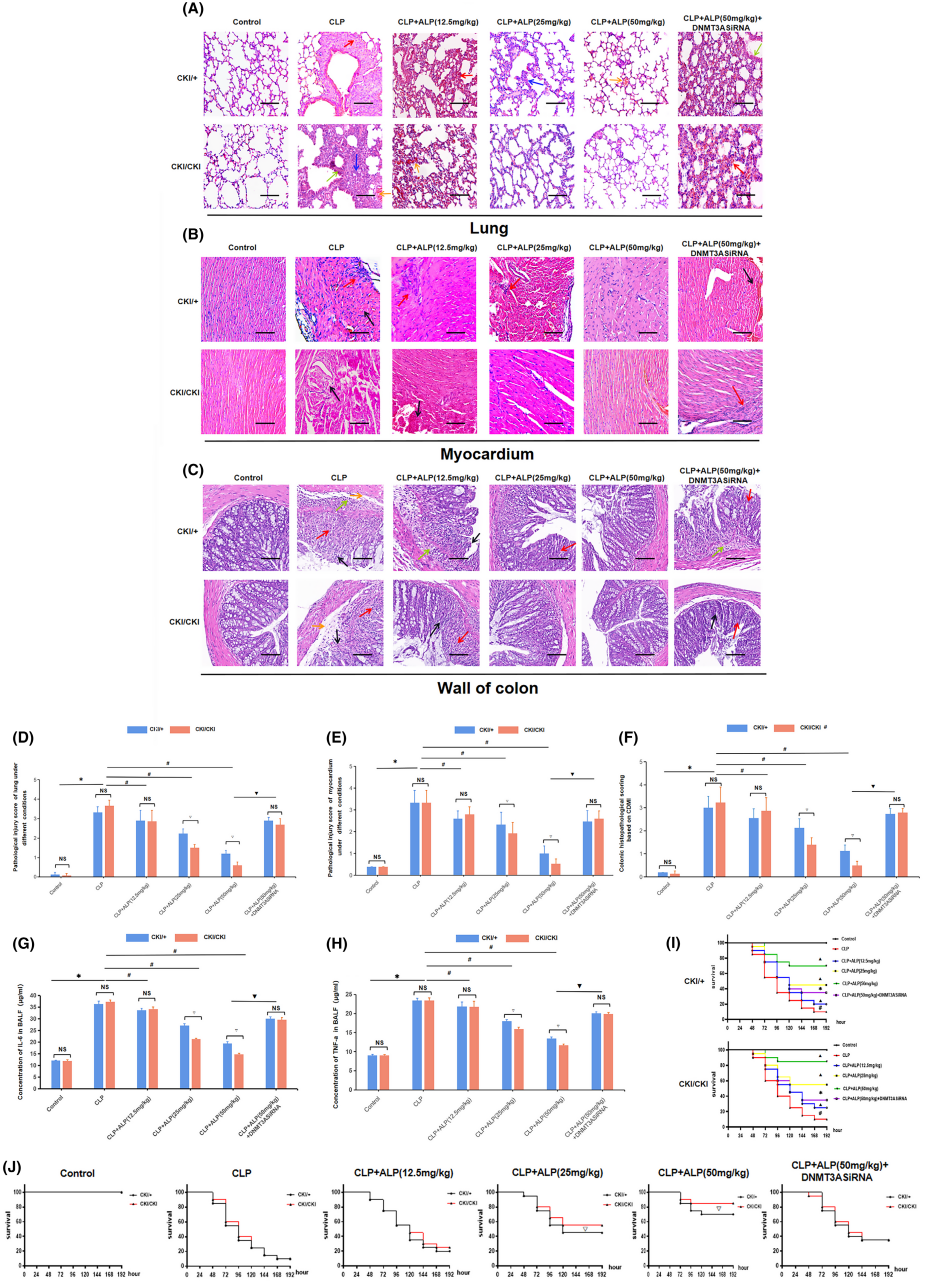
Mecp2 promotes the improvement of alpinetin on sepsis, an inflammatory disorder, and multiple organ injury. (A–C) HE staining of lung, myocardium and colon tissue sections of heterozygous and homozygous objects under various intervention conditions (×100). In histological examination of lung tissue sections, congestion is marked by red arrows, haemorrhage by yellow arrows, neutrophil infiltration by blue arrows and thickening of the alveolar wall by green arrows. In myocardial tissue sections, significant lymphocyte infiltration is indicated by red arrows, while disordered arrangement of the myocardium is denoted by black arrows. Pathological alterations in colon tissue are represented by red, black, yellow and green arrows, indicating absence of goblet cells and crypt structures, edema in the submucosa and infiltration of inflammatory cells, respectively. Scale bar = 100 μm. Pathological injury scores of heterozygous and homozygous objects under various intervention conditions (D–F) and concentration of inflammatory factors in BALF (G, H): *n* = 3, ^✱^ *p* < 0.05 vs. control; ^*#*^ *p* < 0.05 versus CLP, as the intervention concentration of alpinetin gradually increases; ^▼^ *p* < 0.05 versus CLP + ALP (50 mg/kg); ^▽^ *p* < 0.05 versus CKI/+, when heterozygous and homozygous were under the same intervention. (I) Kaplan–Meier analysis comparing the survival curves of heterozygous and homozygous under various intervention conditions: *n* = 20/per group, ^#^ *p* < 0.05 versus control, ^▲^ *p* < 0.05 versus CLP, ^✱^ *p* < 0.05 versus CLP+ ALP (50 mg/kg). (J) Kaplan–Meier analysis comparing the survival curves of heterozygous and homozygous objects under the same intervention conditions: ^▽^ *p* < 0.05 versus CKI/+.

HE staining and pathological scores in each intervention group are shown in Figure 8A–D,F. When no intervention was performed, There were no significant abnormalities in the tissue structure of the lung, myocardium and colon wall. In contrast, the CLP group exhibited notably thickened and broken alveolar walls, fibrotic pulmonary interstitium, damaged and disordered myocardial structure accompanied by invasion of inflammatory cells. Furthermore, significant inflammation also existed in the colon wall, characterized by loss of crypt structure and goblet cells, submucosal edema and significant infiltration of neutrophils. The administration of alpinetin at a concentration exceeding 12.5 mg/kg resulted in a notable reversal of the aforementioned inflammatory pathological damage, where the extent of reversal was directly correlated to the intervention concentration. Also, the variations in organ damage observed under different intervention conditions aligned with the levels of inflammatory factors in BALF and the survival outcomes. Homozygous mice treated with alpinetin (25,50 mg/kg) showed diminished scores of organ pathological damage and lower levels of inflammatory mediator synthesis, resulting in longer survival compared to the heterozygous group (Figure 8D–J). However, research date suggests that if the inhibition of DNMT3A expression occurs, the beneficial effects of alpinetin on the survival rate and inflammatory markers in the CLP model may be negated. Additionally, the disparities in inflammation markers and survival rates between homozygous and heterozygous individuals overexpressing Mecp2 are no longer significant (Figure 8D–J).

## 4 DISCUSSION

With the continuous progress of chromatin immunoprecipitation (Chip‐qPCR) and high‐throughput sequencing (Chip‐seq) detection technology, the epigenetic modification mechanism of inflammation has been gradually confirmed.^21^, ^22^, ^23^, ^24^ For example, reduced methylation levels of H3K27 and increased histone methyltransferase MLL2, G9a and DNMT1 content were proven to exist in atherosclerotic plaque lesions.^25^ Furthermore, activation of the Wnt/β‐catenin pathway induces the reopening of the chromatin structure of the regulatory site of the expression of pro‐inflammatory genes, which leads to the expression of inflammatory upstream activating factors and the progression of ulcerative colitis.^26^ Based on these findings, we hypothesized that a similar mechanism might affect the anti‐inflammatory effect function of alpinetin, a natural flavonoid frequently applied in Chinese patent drugs. Alpinetin can participate in the synthetic intervention process of inflammatory mediators by inducing methylation, deacetylation, and other epigenetic modification effects. In this study, we further explored the mechanism of its anti‐inflammatory effects by focusing on epigenetic modification, precisely, Mecp2, a critical transcriptional regulator capable of binding to methylated DNA. Our data confirmed for the first time that Mecp2 has an assisting function on the anti‐inflammatory effect of alpinetin.

The role of Mecp2 in epigenetic modification has been widely discussed in recent years. Except for nerve tissue, the content of Mecp2 is expressed at low levels in other tissues.^27^ Mecp2 binds to methylated DNA through its methyl‐CpG‐binding domain (MBD), which then inhibits the transcription of genes near the binding site.^28^, ^29^ Many studies supported that Mecp2 can integrate the epigenetic modification effect in organisms, thus mediating the development of inflammation and tumours.^30^, ^31^ For example, mutations in Mecp2 are the predominant cause of Rett syndrome, a disease characterized by neurological symptoms and systemic abnormalities.^32^ Moreover, other studies discovered that Mecp2 regulates susceptibility to experimental colitis by controlling CD11c^+^ cells and colonic epithelium.^33^ In addition, mice with Mecp2 gene mutations seem more prone to inflammatory cell aggregation and structural damage in the alveoli.^34^ However, there is conflicting evidence regarding the ability of Mecp2 to regulate the synthesis of inflammatory mediators. For instance, few studies have demonstrated that overexpression of Mecp2 can amplify the inflammatory response triggered by LPS in BV2 glial cells.^16^ Conversely, our previous study showed a further reduction in the production of inflammatory mediators by macrophages overexpressing Mecp2 when treated with alpinetin. Thus, further investigation is warranted to elucidate the molecular mechanism underlying the role of Mecp2 in facilitating the anti‐inflammatory effect of alpinetin.

Based on the above assumptions, we used macrophages overexpressing Mecp2 and gene‐editing mice to confirm that the combined strategy of alpinetin and Mecp2 positively affects the reversal of uncontrolled inflammation. First, in vitro data confirmed the methylating modification effect of alpinetin mediated by DNA methyltransferase3A (DNMT3A) on the promoter region of inflammatory gene IL‐6. Subsequently, employing ChIP‐seq technology, it was further elucidated that alpinetin may intervene in the synthesis process of inflammatory mediators through two mechanisms: (1) inhibition of the synthesis of classical nuclear factor p65; (2) the methylating modification effect attributed to alpinetin impeded the binding of p65 to the IL‐6 promoter site. Nonetheless, the intervention of alpinetin did not impact the synthesis and binding ability of the co‐activator p300, despite its significant association with LPS stimulation intensity as confirmed by chip analysis. Based on prior research findings, it has been established that p300 can induce the acetylation modification of H3K27, thus facilitating the unfolding of chromatin structure and activating the expression of adjacent genes. Thus, the isolated use of alpinetin may not fully maximize the inhibition of inflammatory mediator synthesis. However, Subsequent Chip‐seq analysis confirmed the presence of a certain quantity of Mecp2 can impede the binding of p300 to the methylated IL‐6 promoter, thereby leading to a reduction in the acetylation modification level of H3K27, which can further inhibit the binding of p65 to the IL‐6 promoter site. The above molecular mechanism leads to a further decrease in the synthesis of inflammatory mediators in macrophages overexpressing Mecp2 compared to non‐overexpressing macrophages in the presence of alpinetin. Furthermore, the elimination of this distinction occurs upon inhibition of DNMT3A, indicating that participation of Mecp2 in the anti‐inflammatory efficacy of alpinetin is facilitated by its association with the methylated promoter. The above results was further confirmed through gene manipulation studies in animals, where homozygous mice treated with alpinetin exhibited reduced inflammatory damage in lung, myocardial, and colon tissues, as well as an increased survival rate compared to heterozygous mice. The findings suggest that the suppressive ‘crosstalk’ effect on acetylated modification induced by p300 plays a significant role in the molecular mechanism of Mecp2, thereby facilitating the anti‐inflammatory properties of alpinetin.

Considering the strong affinity of Mecp2 for methylated promoters and its restricted expression in immune cells, a more effective approach for treating inflammatory disorders may involve a combination of increasing Mecp2 expression and administering alpinetin. This integrated treatment strategy aligns with the concept of merging traditional Chinese and Western medicine practices. However, the present study also has some limitations. First, as a widely used methyltransferase in the biological organism, many genes whose regulation is affected by CpG island are interrupted by DNMT3A; hence, it is imperative to verify whether the activation of DNMT3A by alpinetin influences the methylation status of additional CpG islands and whether such modifications result in detrimental effects. Second, although no negative effects on growth and cognitive behavioural performance in mice have been observed as a result of Mecp2 overexpression, it is still uncertain whether this approach is equally safe over a prolonged period due to the limited duration of observation. Third, the considerable expression of Mecp2 in neural tissues warrants additional investigation into the potential efficacy of a combined approach involving Mecp2 and alpinetin in suppressing neuro‐inflammatory responses, consequently impeding the advancement of neurodegenerative disorders such as Alzheimer's and Parkinson's disease. Additionally, further exploring methods for achieving stable and secure overexpression of Mecp2 in human subjects is needed.

## 5 CONCLUSION

**FIGURE 9:**
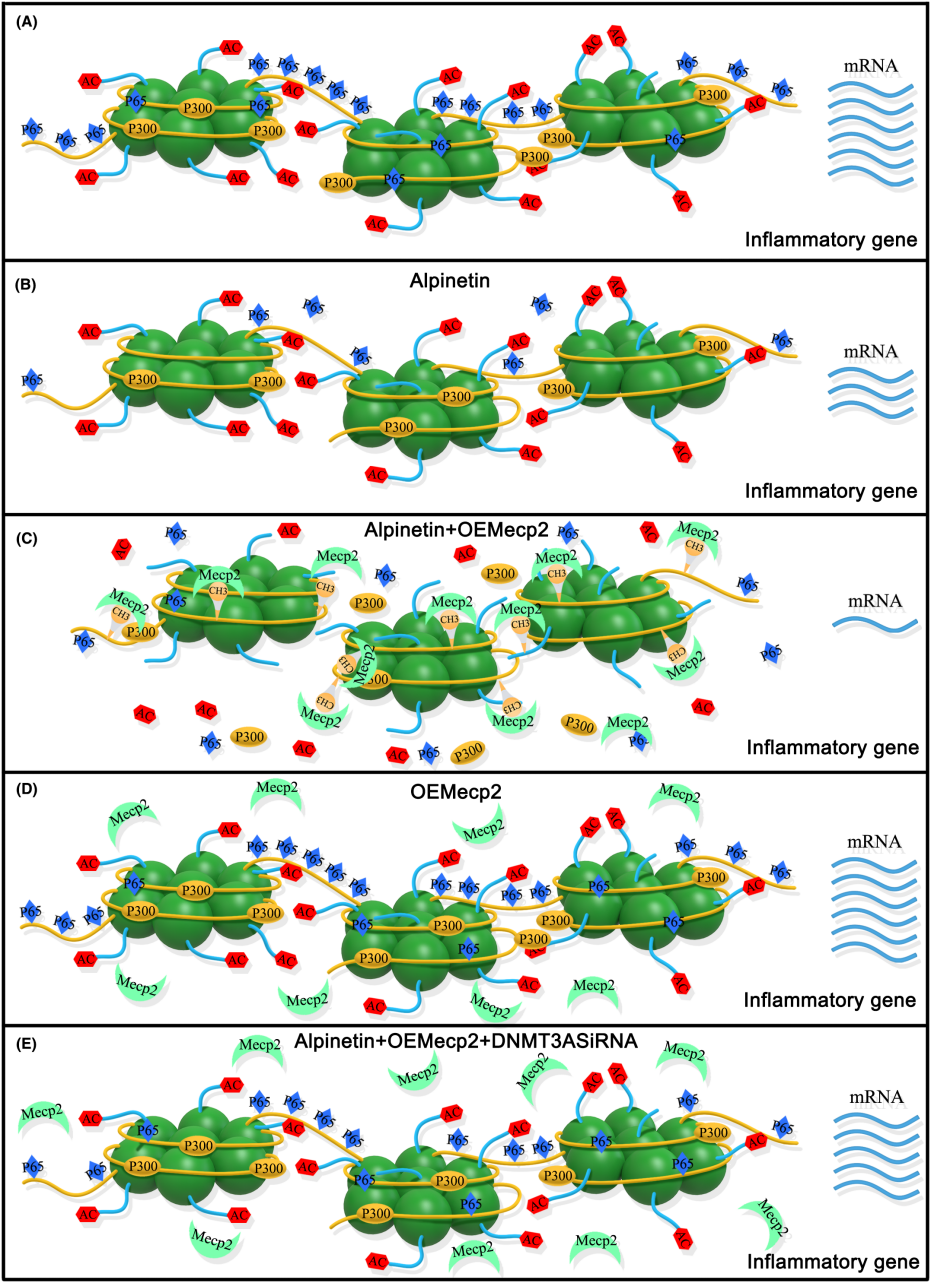
The molecular mechanism of Mecp2 assisting the anti‐inflammatory effect of alpinetin. (A) According to the research results, it can be concluded that both p65 (a member of the nuclear factor family) and p300 (coactivator) dominate the synthesis process of inflammatory mediators. However, the dependent mechanisms of p65 and p300 are completely different: p65 is a direct transcription activator, while the transcriptional activation effect of p300 depends on its acetylated modification of H3K27, which relaxes the chromatin structure and makes p65 easier to combine. (B) The inhibition of alpinetin on the synthesis of inflammatory mediators is realized through two mechanisms: (1) direct inhibition of p65 synthesis; (2) the methylated effect on the promoter of the inflammatory gene, which further affects the binding of p65. However, alpinetin cannot interfere with the synthesis of p300 or its acetylated modification effect. (C) If alpinetin and Mecp2 coexist, the latter can affect the binding of p300 via combining with the methylated promoter, reducing the acetylated modification level of H3K27 and further reducing p65 binding. Therefore, Mecp2 plays a role of ‘crosstalk’, which links the methylated modification caused by alpinetin and the acetylated modification associated with p300 to act on the regulation of gene expression involved in inflammation, resulting in that compared with alpinetin alone, the combination of alpinetin and Mecp2 can further inhibit the synthesis of inflammatory mediators. (D, E) Consequently, in the absence of alpinetin or in the presence of alpinetin with blocked DNMT3A, Mecp2 is unable to bind to non‐methylated promoter fragments, resulting in the loss of its regulatory influence on the expression of inflammatory mediators. To sum up, the combined strategy of Mecp2 and alpinetin may be a promising intervention plan for inflammatory disorders.

The inhibition of the interaction between p300 and gene fragments can potentially impact the extent of acetylated modification, a crucial mechanism through which Mecp2 regulates gene expression and aids in the anti‐inflammatory properties of alpinetin (Figure 9). These findings suggest that the combined use of Mecp2 and alpinetin could be a viable therapeutic option for managing inflammatory disorders, such as multiple organ damage associated with sepsis.

## AUTHOR CONTRIBUTIONS

**Ke Hu:** Conceptualization (lead); data curation (equal); funding acquisition (equal); methodology (equal). **Bifeng Tan:** Conceptualization (lead); funding acquisition (lead); methodology (equal); project administration (equal). **Huiming Yin:** Conceptualization (lead); funding acquisition (equal); investigation (equal); project administration (equal). **Ruoting Ma:** Conceptualization (equal); data curation (lead); methodology (lead); project administration (equal). **Minjiang Huang:** Conceptualization (equal); investigation (equal); methodology (equal); project administration (equal). **Xiangyu Cao:** Data curation (lead); investigation (equal); methodology (equal). **Sha Ling:** Conceptualization (equal); data curation (equal); investigation (equal). **Yuefu Chen:** Conceptualization (equal); investigation (equal); methodology (equal). **Yuxian Li:** Conceptualization (equal); data curation (equal); formal analysis (equal); methodology (equal). **Yan Ding:** Conceptualization (equal); formal analysis (equal); methodology (equal). **Lijun Xiao:** Data curation (equal); investigation (equal); methodology (equal). **Youliang Huang:** Conceptualization (equal); formal analysis (equal); methodology (equal).

## FUNDING INFORMATION

This work was supported by the National Natural Science Foundation of China (NO. 82070081), Joint project of Hunan Provincial Natural Science Foundation (NO. 2024 JJ7321) and the project of clinical medical technology demonstration base for hypertension prevention and treatment in Huaihua city, Hunan province (NO. 2023N2404).

## CONFLICT OF INTEREST STATEMENT

The authors declare that they have no competing interests.

## Data Availability

All data generated or analysed during this study are included in this published article.
